# A New Multi-Sensor Fusion Scheme to Improve the Accuracy of Knee Flexion Kinematics for Functional Rehabilitation Movements

**DOI:** 10.3390/s16111914

**Published:** 2016-11-15

**Authors:** Halim Tannous, Dan Istrate, Aziz Benlarbi-Delai, Julien Sarrazin, Didier Gamet, Marie Christine Ho Ba Tho, Tien Tuan Dao

**Affiliations:** 1 Sorbonne University, Université de Technologie de Compiègne, CNRS, UMR 7338 Biomechanics and Bioengineering, 60200 Compiègne, France; mircea-dan.istrate@utc.fr (D.I.); didier.gamet@utc.fr (D.G.); marie-christine.ho-ba-tho@utc.fr (M.C.H.B.T.); tien-tuan.dao@utc.fr (T.T.D.); 2 Sorbonne University, Université Pierre et Marie Curie, L2E, Place Jussieu, 75252 Paris, France; aziz.benlarbi_delai@upmc.fr (A.B.-D.); julien.sarrazin@upmc.fr (J.S.)

**Keywords:** multi-sensor fusion, orientation estimation, home-based functional rehabilitation, exergames

## Abstract

Exergames have been proposed as a potential tool to improve the current practice of musculoskeletal rehabilitation. Inertial or optical motion capture sensors are commonly used to track the subject’s movements. However, the use of these motion capture tools suffers from the lack of accuracy in estimating joint angles, which could lead to wrong data interpretation. In this study, we proposed a real time quaternion-based fusion scheme, based on the extended Kalman filter, between inertial and visual motion capture sensors, to improve the estimation accuracy of joint angles. The fusion outcome was compared to angles measured using a goniometer. The fusion output shows a better estimation, when compared to inertial measurement units and Kinect outputs. We noted a smaller error (3.96°) compared to the one obtained using inertial sensors (5.04°). The proposed multi-sensor fusion system is therefore accurate enough to be applied, in future works, to our serious game for musculoskeletal rehabilitation.

## 1. Introduction

In the past few decades the growing population has underlined many healthcare problems. According to the World Health Organization, 15% of people currently suffer from musculoskeletal disabilities. Among these 15%, 35%–50% of disabled people in modern countries are not getting the necessary management (diagnosis, treatment, follow-up). This number is even higher in developing countries and reaches 76%–85% [1]. Functional rehabilitation is one of the most efficient routine practices to recover any dysfunction of body locomotion, and to improve surgical outcomes of musculoskeletal patients. Conventionally, a rehabilitation program is assigned by a clinical expert to a specific patient and then the execution and follow-up are managed by a team of therapists. This approach, known as direct therapist intervention, presents some limitations that require therapists to always follow, guide, and physically support their patients [2]. A significant number of therapists is required to ensure the quality of the rehabilitation program. Thus, the cost and time consumptions of the therapy sessions, for both patients and therapists, are the biggest disadvantages of this approach. Moreover, other negative factors relate to the lack of self-motivation for patient due to the repetitiveness of the assigned exercises. These facts led to the establishment of a new research field known as home based rehabilitation, as a complementary tool for therapeutic sessions. Furthermore, a new rehabilitation approach, known as exergames [3,4,5,6,7,8,9,10,11,12,13,14,15], has been proposed to help motivate patients while performing their rehabilitation tasks at home. This new concept incorporates games into the rehabilitation process, to add a motivational and challenging aspect to these programs. Exergames have shown their clinical relevance in improving exercise execution, body equilibrium, joint flexibility, and muscle strength.

A large range of sensors like the Microsoft Kinect, Wii Mote, Wii Fit, force plates, and inertial measurement units (IMU) have been used as interactive tools between the subject and the virtual environment of the developed exergaming systems [3,4,8,9,10,11,12]. The most commonly used sensor is the Microsoft Kinect, due to its low price and big success with Xbox games. In order to use these visual or inertial sensors for body tracking in serious games, the sensor needs to be able to estimate the orientation of any considered limb and joint angles. Several tools can help estimate these parameters. The universal goniometer was the most famous tool for estimating joint angles, and more recently, the VICON motion capture system is commonly used for the same purpose. However, even though these two tools are considered as the golden standards for orientation and angle estimation, they are neither portable nor cost efficient. This led to a growing interest in using IMUs in particular.

Several researchers were interested in exploiting IMUs for this purpose. Williamson et al. used two biaxial accelerometers and two uniaxial gyroscopes, attached to the subject’s thigh and shank, to determine the knee angle using several algorithms [16]. The angle estimation was compared to a universal goniometer, and the results showed that the algorithm integrating the gyroscope’s angular velocity and automatically nulling the angular velocity integrator, using the accelerometer data, was the closest to the knee angle measured using a goniometer. Myagoitia et al. [17] obtained good results when they calculated the angles and angular velocities using two uniaxial gyroscopes, and the linear and angular acceleration using four uniaxial accelerometers, with one gyroscope and two accelerometers on the thigh and shank, respectively. The results were compared to those measured using the universal goniometer. Favre et al. then explained that there is an error due to the difference in the alignment of the sensors references [18]. They developed an algorithm that considers this problem using two three dimensional (3D) accelerometers and two 3D gyroscopes, and achieved good results for knee angle calculation with respect to a golden standard. Recent advances in integrated circuits have led to a higher availability of IMU’s and thus research on this topic has flourished. Liu et al. developed their own IMU using biaxial accelerometers and 3D gyroscopes [19]. After combining data from both sensors and correcting the measurement based on a prior calibration, they achieved a root mean square error (RMS) of about 5° for knee angle estimation compared to an optical reference. Perez et al. studied a commercially available IMU for upper limb orientation estimation [20]. The IMUs calculated the Euler angles internally, and the researchers transformed these angles to quaternions to estimate joint angles between sensors. They compared their outputs with a visual motion tracking reference and found inaccurate results for angle estimation. However, they applied calibration for one sensor to determine shoulder internal external rotation, and obtained a good RMS of 0.8° using one sensor and a simple gesture.

The use of filtering technics for orientation estimation began when Marins et al. proposed an algorithm using Extended Kalman filter [21] for quaternion-based orientation estimation using an IMU with tri-axis accelerometer, gyroscope, and magnetometer [22]. The results of the orientation estimation were not compared to a reference; however, the team studied the convergence of the measured quaternions. In addition, Abayarjoo et al. used a similar IMU with a linear Kalman filter to determine the angle directly from the sensor, without passing through a quaternion analysis [23]. The proposed algorithm helped to overcome the limitations of the accelerometer, because accelerometers generally measure two components: an acceleration and a gravitational component. The acceleration component needs to be eliminated to determine the orientation, and thus Kalman filtering with gyroscopes was presented as the best solution. Madgwick et al. studied a new type of algorithm based on gradient descent using quaternions, in order to fuse tri-axial accelerometers, gyroscopes and magnetometers [24]. They compared their algorithm and the extended Kalman filter approach with a reference optical measurement system and proved that the new algorithm was better than the older approach in both static and dynamic cases. Later on, Madwick’s algorithm was implemented in many commercially available IMUs, e.g., Shimmer 9DoF sensors [25]. Miezal et al. proposed two new extended Kalman filters and a sliding window optimization approach to estimate upper limb joint angles [26]. The authors also studied the effect of sensor to segment calibration through the use of simulated data. The study concluded that the proposed methods were more efficient in estimating upper limb joint angles, with the sliding optimization approach being the best algorithm among them.

Some studies were interested in calibrating IMUs to the body of each subject. Dejnabadi et al. focused their work on trying to find the accurate knee, hip, and shank angles with respect to the subject’s personalized musculoskeletal system [27,28]. Their idea was that using sensors attached directly to the patient will not yield the correct joint angles, so they used two biaxial accelerometers, two uniaxial gyroscopes, and a two dimensional image of the subject, to calculate the joint angles. They found good results compared to a golden standard reference system (1.57° error for thigh angle and 0.78° error for shank angle for subjects walking at medium speed with a low range of motion). Favre et al. proposed, in a study following [18], to move the joint angle computation to a personalized musculoskeletal model for each subject, using calculations prior to the measurements [29], and obtained better results. Bouvier et al. studied the effect of applying different sensor-to-segment calibration method on upper limb kinematics [30]. They applied three different calibration techniques, and used 10 subjects for their study. They concluded that all three methods showed similar results (a range of 5 to 10 degrees of error).

The Kinect presents a different situation, and only a few researchers have studied algorithms for orientation estimation, since the manufacturer already provided a tool to compute the orientation of body joints. Zhang et al. used multiple Kinect cameras to estimate the position of a subject [31]. Some recent studies were interested in combining the Kinect and IMUs, these studies are presented in Table 1.

Feng et al. used a linear multi-rate Kalman filter implanted to compute the position of some joints using data from both the Kinect and IMUs [32]. The study resulted in a better estimation of the positions of joints compared to a reference system. Another study done by Destelle et al. tried to use Kinect first to determine the initial positions and then calculate the positions and angles of the joints using IMUs [33]. Atrsaei et al. published a study where they proposed a fusion algorithm between the Kinect and inertial sensors using an unscented Kalman filter, applied to the upper body [34]. The suggested method was efficient in reducing the error of joint position calculation, however, the orientation estimation precision did not improve significantly. Kalkbrenner et al. used a linear Kalman filter to estimate joint positions, using unit orientation vectors acquired from IMUs and joint positions given by the Kinect [35]. The study used 10 subjects to validate their method, but did not compare the joint positions with a reference system. Tian et al. proposed an unscented Kalman filter for fusion between IMUs and the Kinect camera [36]. They compared the estimated joint positions with a reference system, but did not study the error of the elbow angle estimation. Finally, Glonek et al. proposed a joint position and angle estimation method, based on averaging inputs from the Kinect and IMU sensors [37]. The study was validated with one subject, performing exercises with different ranges of motion. Therefore, there is still a lack of efficient orientation estimation techniques based on a fusion between the Kinect and IMU.

The objective of the present paper is to develop and validate an orientation-based fusion scheme between visual and inertial sensors to improve the knee flexion kinematics during functional rehabilitation movement of the lower limbs. An extended Kalman filter will be used as a fusion technique between the Kinect and IMU sensors. The knee flexion was chosen for this study since our previous works showed that some exergames cannot be assessed due to a lack of accuracy in estimating this particular angle [4,38].

## 2. Materials and Methods

### 2.1. Orientation-Based Multi-Sensor Fusion Scheme

**Figure 1:**
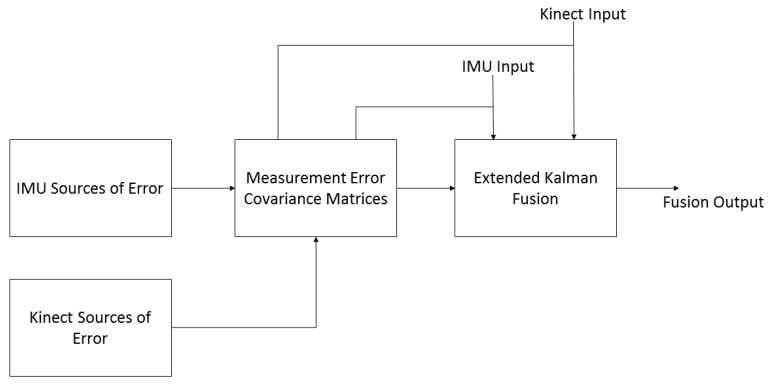
Schematic illustration of the developed orientation-based multi-sensor fusion scheme.

A real time, quaternion based, extended Kalman filter was developed to fuse the outcomes of one Kinect visual sensor and two Shimmer IMU sensors. The overview of our developed fusion scheme is shown in Figure 1.

The estimation of the measurement noise covariance matrices is performed using the results of the Kinect and IMU sources of errors analysis. Then, these matrices are integrated into an extended Kalman algorithm with Kinect and IMU signals to estimate the knee joint kinematics in real time conditions. Each component of the proposed multi-sensor fusion scheme is detailed in the following sections.

### 2.2. Computing of Measurement Noise Covariance Matrices

The state vector in our Kalman filter fusion scheme is composed of quaternions and angular velocities relative to the knee angle (i.e., angle defined between shank and thigh). The covariance matrix of measurement noise is a 7 × 7 diagonal matrix, since the measurement vector has seven elements (four quaternion and three angular velocity components). The quaternion error components are estimated from the knee angle estimation and knee angle errors using transformations in [39]. We supposed that the error of estimation for the three angles, between two sensors or two segments for the Kinect, is the same in all dimensions. Thus, the calculated error on knee angles is the same for the three Euler angles. We then took the quaternion calculation formula from Euler angles and partially derived those formulas to obtain the following equations:

(1) $$R_{1}={\left(\frac{\Delta \varphi }{2}\left[\left(-\mathrm{Cos}\left[\frac{\psi }{2}\right]+\mathrm{Sin}\left[\frac{\psi }{2}\right]\right)\left(\mathrm{Sin}\left[\frac{\theta }{2}\right]\mathrm{Cos}\left[\frac{\varphi }{2}\right]+\mathrm{Sin}\left[\frac{\varphi }{2}\right]\mathrm{Cos}\left[\frac{\theta }{2}\right]\right)-\mathrm{Cos}\left[\frac{\theta }{2}\right]\mathrm{Cos}\left[\frac{\varphi }{2}\right]\mathrm{Sin}\left[\frac{\psi }{2}\right]+\mathrm{Cos}\left[\frac{\psi }{2}\right]\mathrm{Sin}\left[\frac{\theta }{2}\right]\mathrm{Sin}\left[\frac{\varphi }{2}\right]\right]\right)}^{2}$$

(2) $$R_{2}={\left(\frac{\Delta \varphi }{2}\left[\left(\mathrm{Cos}\left[\frac{\psi }{2}\right]-\mathrm{Sin}\left[\frac{\psi }{2}\right]\right)\left(\mathrm{Cos}\left[\frac{\theta }{2}\right]\mathrm{Cos}\left[\frac{\varphi }{2}\right]-\mathrm{Sin}\left[\frac{\varphi }{2}\right]\mathrm{Sin}\left[\frac{\theta }{2}\right]\right)-\mathrm{Cos}\left[\frac{\theta }{2}\right]\mathrm{Sin}\left[\frac{\varphi }{2}\right]\mathrm{Sin}\left[\frac{\psi }{2}\right]-\mathrm{Cos}\left[\frac{\psi }{2}\right]\mathrm{Sin}\left[\frac{\theta }{2}\right]\mathrm{Cos}\left[\frac{\varphi }{2}\right]\right]\right)}^{2}$$

(3) $$R_{3}={\left(\frac{\Delta \varphi }{2}\left[\left(\mathrm{Cos}\left[\frac{\psi }{2}\right]+\mathrm{Sin}\left[\frac{\psi }{2}\right]\right)\left(\mathrm{Cos}\left[\frac{\theta }{2}\right]\mathrm{Cos}\left[\frac{\varphi }{2}\right]-\mathrm{Sin}\left[\frac{\varphi }{2}\right]\mathrm{Sin}\left[\frac{\theta }{2}\right]\right)-\mathrm{Sin}\left[\frac{\theta }{2}\right]\mathrm{Cos}\left[\frac{\varphi }{2}\right]\mathrm{Sin}\left[\frac{\psi }{2}\right]+\mathrm{Cos}\left[\frac{\psi }{2}\right]\mathrm{Cos}\left[\frac{\theta }{2}\right]\mathrm{Sin}\left[\frac{\varphi }{2}\right]\right]\right)}^{2}$$

(4) $$R_{4}={\left(\frac{\Delta \varphi }{2}\left[\left(\mathrm{Cos}\left[\frac{\psi }{2}\right]+\mathrm{Sin}\left[\frac{\psi }{2}\right]\right)\left(-\mathrm{Cos}\left[\frac{\theta }{2}\right]\mathrm{Sin}\left[\frac{\varphi }{2}\right]-\mathrm{Cos}\left[\frac{\varphi }{2}\right]\mathrm{Sin}\left[\frac{\theta }{2}\right]\right)+\mathrm{Cos}\left[\frac{\theta }{2}\right]\mathrm{Cos}\left[\frac{\varphi }{2}\right]\mathrm{Cos}\left[\frac{\psi }{2}\right]+\mathrm{Sin}\left[\frac{\psi }{2}\right]\mathrm{Sin}\left[\frac{\theta }{2}\right]\mathrm{Sin}\left[\frac{\varphi }{2}\right]\right]\right)}^{2}$$

where θ is the knee pitch angle, ψ the knee yaw angle, ϕ the knee roll angle, and ∆ϕ the error calculated on roll angle considered similar to errors in pitch and yaw angles. The value of ∆ϕ varies between Kinect and Shimmer sensors and is determined from the results of the analysis of the sources of errors of these sensors. We consider that the knee does not have a yaw component, relative to the thigh, since the knee joint cannot execute an internal external rotation motion when considering the high knees exercise adopted for our test. Consequently, the term ψ is taken equal to 0 for the remaining of the paper. The measurement noise matrix is dynamically calculated at each step of the Kalman filter since it needs the value of the current knee pitch and roll angles. For the rest of the diagonal values, related to angular velocities, Shimmer manufacturers have given a description of the sensor’s gyroscopes accuracy [25], and the Kinect’s accuracy was calculated from the root mean square (RMS) error of the angular velocity.

#### 2.2.1. IMU Sources of Error

**Figure 2:**
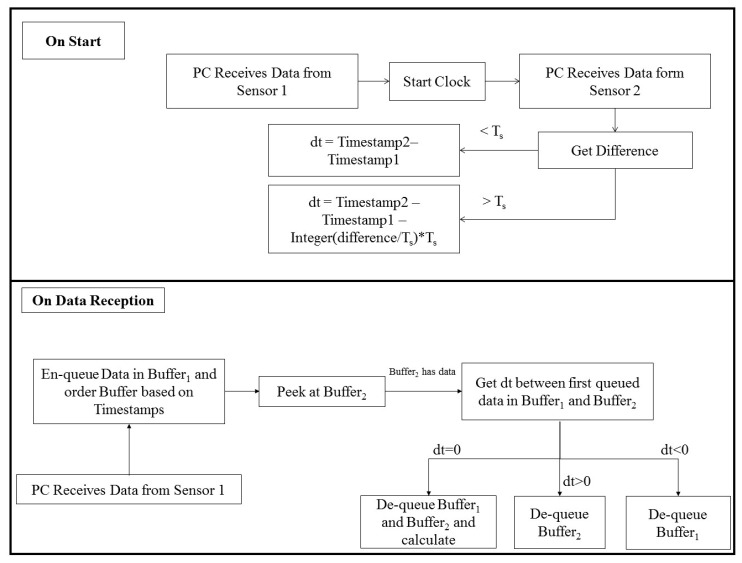
Data flow of sensor synchronization algorithm.

To estimate the error of an IMU, three main sources of error are analyzed and quantified: sensor synchronization, orientation estimation algorithm, and sensor displacement due to muscle artefacts [40]. One of the leading sources of error, when coupling two inertial sensors at the same frequency rate, is caused by the lack of synchronization. The Shimmer sensor Application Programming Interface (API) does not give access to a universal clock measurement from each sensor, and thus, synchronization is not an easy task to perform. The sensor also streams data continuously and without waiting for requests from the Personal Computer (PC), and synchronization methods similar to those propose in [41] cannot be applied. However, we did have access to a local clock from each sensor, which starts the count at each program start, and so we proposed the following synchronization algorithm. The data flow of our developed real time synchronization method is shown in Figure 2. Let us consider two Shimmer sensors communicating with a PC via a Bluetooth module at a sampling rate of $f_{s}=\frac{1}{Ts}\;=51.2\;\mathrm{Hz}$. These sensors send data constantly at each multiple of $T_{s}$, without a data request message from the PC. The data includes sensor measurements and a local timestamp that indicates sending time (which can be interpreted as a counter that indicates the number of the sent sample). The two sensors start to stream data after receiving a “StartStreaming” request message from the PC. dt is the tested quantity that will be measured between two samples sent from the two sensors, and that indicates their compatibility if it’s value is equal to zero. Let us consider that sensor 1 sends the first sample. The program then initializes a local clock on the PC, waits for the first sample from sensor 2, then computes the time difference between the two samples in the PC’s local time. If the difference is lower than $T_{s\;}$, the value dt that will be computed at each sample reception will be the difference between the two local timestamps of two samples from the two sensors. If it is higher, dt will include a component based on the first delay between the two sensors, measured by the PC. Then, when the PC receives a sample from any sensor, it puts the value inside a specific buffer and orders the buffer by increasing timestamp, then checks if the buffer of the other sensor has any data. If data exists in the buffer of the other sensor, and dt between the first sample in each of the two buffers is null, we de-queue both buffers and calculate. If not, then there is a loss in data from either sensor 1 or 2, so we de-queue one of the buffers based on the value of dt and the current thread. The case of sensor 1 is presented in Figure 2.

**Figure 3:**
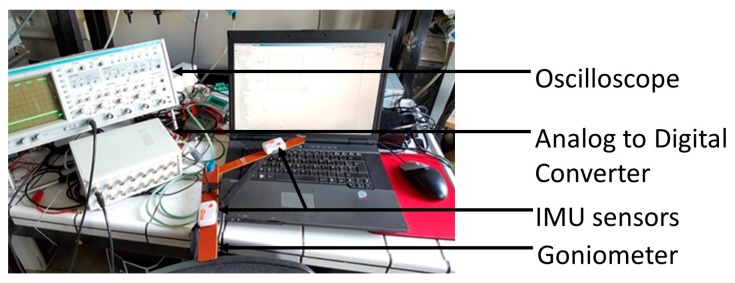
Goniometer protocol set-up for testing.

Extended Kalman [22] and Gradient Descent [24] algorithms are used to estimate the sensor orientation. To assess the accuracy of the estimation, we compare the angle between two inertial sensors, with a universal goniometer, while varying the velocity of the angle movement between three states: fast, slow, and a combination (fast followed by slow). The sensors are mounted directly on the goniometer to prevent sensor displacement due to muscular flexion or extension. The test consists in repeatedly moving the goniometer’s arms closer then farther. Three trials are conducted at each speed, with and without application of our synchronization algorithm. Figure 3 shows the material used during this test.

**Figure 4:**
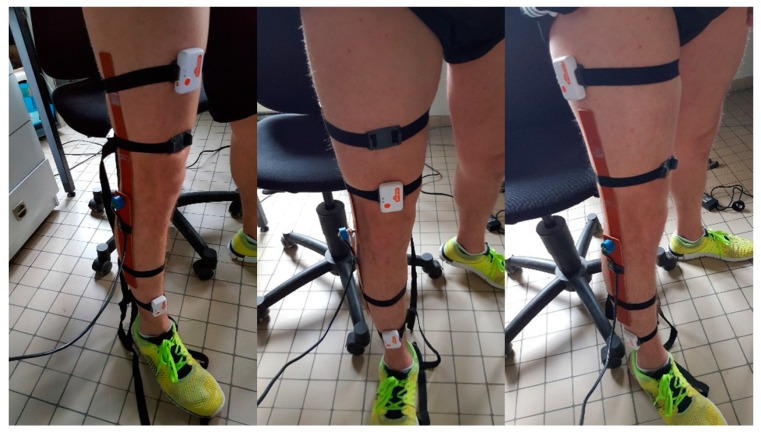
Tested sensor positions (**Left**: Sensor on the thigh directly; **Middle**: Sensor above the kneecap; **Right**: Sensor in the sagittal plane).

The position of sensors on the thigh and shank is an important aspect since it is affected by artefacts due to muscle flexion and extension, and tissue displacement. One healthy subject was chosen for this test (male, 23 years old, 177 cm body height, 70 kg body weight, and 22.3 kg/m^2^ body mass index (BMI)), in order to estimate the value of ∆ϕ (Section 2.2) to serve as constant for the measurement noise matrix. This subject signed an informed consent agreement before participating in the evaluation process. The high knees exercise was used as a testing movement. We varied the position of the sensors on the thigh and shank in order to study the best possible position to place them. To do so, we tested three different positions, based on previous works in gait measurement [42], and activity detection [43], and then compared the three estimated knee flexion angles with those measured from the universal goniometer as shown in Figure 4. Each test was repeated three times to ensure the reproducibility of the error estimation. The goniometer was adjusted so that the connecting pin, between both segments, is aligned on the knee joint and does not move when executing the high knee movement.

#### 2.2.2. Kinect-Based Sources of Error

Using the Kinect does not give the user the possibility to change parameters in the orientation estimation algorithm. The camera uses a quaternion based algorithm to estimate the quaternion values for each bone. This leads to one unique error source integrated from the camera itself, and out of our control. Therefore, we compared the result of Kinect knee flexion estimation algorithm directly to that of the universal goniometer, in order to obtain RMS values of the error of its angle and angular velocity estimation (derivation of the knee flexion angle). Three high knees trials were also conducted using Kinect and the goniometer, performed by the healthy subject described in Section 2.2.1.

### 2.3. Data Fusion

**Figure 5:**
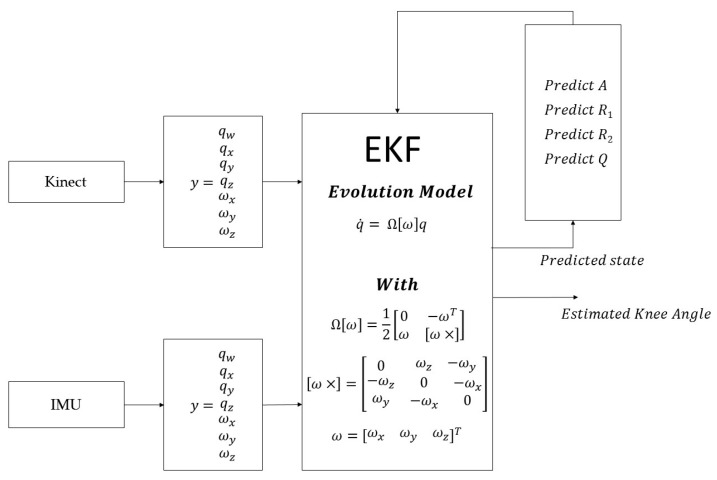
Quaternion based extended Kalman observer scheme for fusion.

Finally, after determining all of our pre-required parameters (angle and angular velocity estimation errors from IMUs and Kinect) for data fusion, we adopted the following scheme shown in Figure 5. Note that these parameters are calibrated with only one subject due to their little effect on the fusion outcome, if they varied in small degrees. We obtained the range of value for ten tested subjects and found that this range is similar to the range obtained with one subject. Precisely, the mean error for IMU placement was within the margin of error of the values taken from one subject for all sensor positions. The same can be stated for the Kinect sensor. Thus, we decided to use this information for all subjects to perform the sensor fusion in real time conditions. This will help avoid additional tests on each subject when the data fusion is applied.

The input from each separate source is the vector *y*, the state vector is composed from the four components of the normalized quaternion and three components of the angular velocity. The sources are treated in the extended Kalman filters using the evolution model shown in Figure 5. After each Kalman step, the predicted state is used to update the measurement noise covariance matrices (*R*_1_ and *R*_2_), the update matrix (*A*) and the process noise covariance matrix (*Q*) which was calculated through equations presented in [44]. The state quaternion was normalized after each step to avoid any problems related to the quaternion unit length. The Kinect frequency (30 Hz) was adopted for the fusion algorithm, for several reasons. On one hand, the Kinect frequency was enough to assess the exercises that were developed in our previous work [4]. On the other hand, in order to keep a real time aspect for our system, we avoided recording data from both sensors, and interpolating the data from the Kinect at IMU sample reception (50 Hz) in an offline analysis. Finally, to assess the synchronization between the two systems, at each Kinect sample reception, we chose the synchronized samples, from both IMUs, that are the closest to the received Kinect sample.

### 2.4. Accuracy Analysis

The proposed real time quaternion-based extended Kalman filter was tested on 10 healthy subjects (mean age 25.4 ± 3.30, mean height 178.2 ± 5.35 and mean weight 75.8 ± 11.58). Each subject signed an informed consent agreement before participating in the evaluation process. The high knees exercise was used as a testing movement, and three trials were performed on each subject for each sensor position, which amounts to nine trials per subject. Note that to test the developed real time synchronization algorithm, we computed the value of dt with synchronization and the difference between two received timestamp from different sensors without synchronization. Finally, the outcome of the fusion algorithm was evaluated against the goniometer measurement. The output signals were aligned, during our offline analysis, so that the correlation between each two signals is at its maximum level.

## 3. Results

### 3.1. Synchronization

The synchronization algorithm prevents data with different timestamps to be coupled with each other. During the experiments, we did not obtain any time difference between the coupled samples of the two sensors. However, without synchronization, differences between timestamps varied between ±100 ms. Furthermore, the variability between the synchronized and not-synchronized angle estimation is 7.44°. Finally, the sensitivity of knee flexion angle estimation at high speeds is around 0.492°/ms (computed as the tangent of the knee flexion angle). In other words, if a person is rotating the knee at a high speed, a difference of one timestamp between samples from separate sensors can lead to an error of 9.6° in the estimation of the knee angle.

### 3.2. Orientation Estimation Algorithm

The outcomes of the three trials of the synchronized (synced for abbreviation) and not-synchronized (not synced for abbreviation) algorithms against the goniometer measurements are presented in Table 2. The root mean square (RMS) difference between the knee angle estimated by the algorithms and measured by the goniometer was calculated, as well as the correlation coefficient (CC) (mean, std, max, min derived from three experiments). The results showed that a higher speed configuration led to a higher RMS error for all of our tested algorithms. This phenomenon was observed when considering the Slow speed data in the test with Fast then Slow movement, which yielded values close to those obtained from the Slow test. Furthermore, a difference of RMS was observed between synced and not synced outputs, e.g., the Gradient Descent output showed a mean RMS error of 3.246° at slow speeds without synchronization and a mean RMS error of 1.8057° with synchronization. Moreover, the synchronization provided accurate data outcome according to goniometer output, especially at high speeds. Furthermore, the CC of the outputs of algorithms without synchronization decreased at higher speeds, while the synchronized algorithms were less affected by the same factor. The Gradient Descent Synced algorithm also yielded the best mean, std, max and min RMS error for all of the tested speeds, while the extended Kalman not synced algorithm gave the worst. Thus, Gradient Descent Synced algorithm was selected for further experiments.

### 3.3. Sensor Position

Table 3 shows the results (RMS and CC) obtained with the different tested sensor positions using the Gradient Descent Synced algorithm.

The sensor placed on the muscle led to the highest values of error (mean RMS = 8.03°) while the other two positions exhibited better performance: mean RMS error in frontal plane is equal to 4.75° while mean RMS deviation in sagittal plane is equal to 4.48°. All the positions show high correlations with the goniometer output. The mean RMS values were used as constant parameters in our fusion filter for later trials.

### 3.4. Kinect Measurement Error

The Kinect’s measurement error is presented in Table 4. The RMS and correlation coefficient of the angle and angular velocity were calculated with respect to the goniometer. The precision of the Kinect camera for calculating knee angles is very poor compared to that of IMUs. The mean RMS error of angle estimation is 14.65° compared to a 4.48° error using the Gradient Descent algorithm. However, a high correlation is achieved between the estimated angle using the Kinect and the one measured by the goniometer (mean CC = 0.974). The angular velocity yielded a 1.33°/s mean RMS error. These values are used later as inputs for our fusion algorithm.

### 3.5. Data Fusion

**Figure 6:**
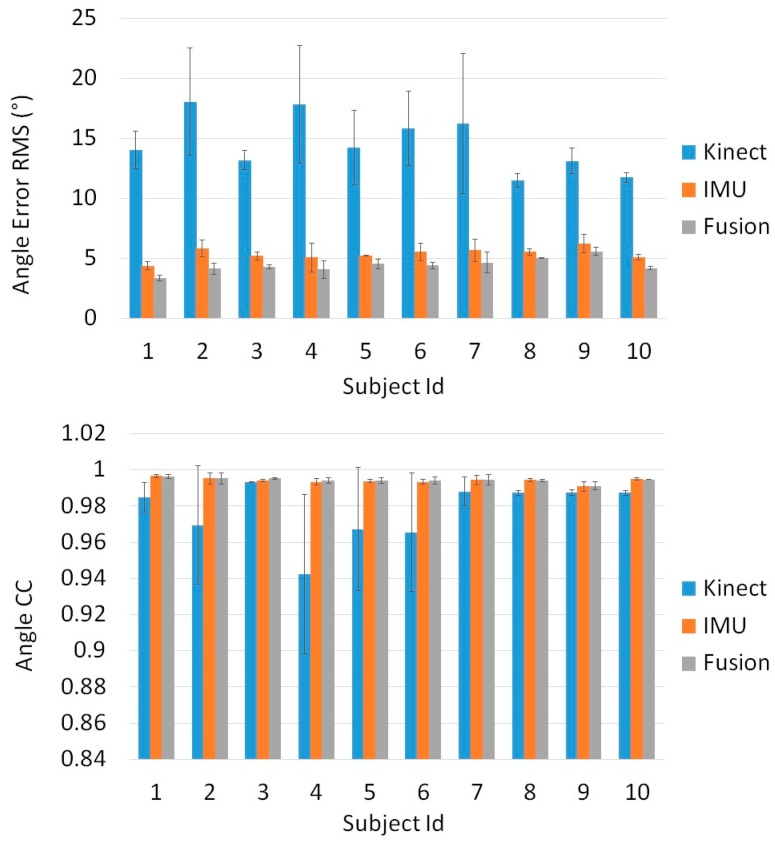
Angle error RMS (°) and angle Correlation Coefficient calculated for the Kinect, IMU and the proposed fusion algorithm Vs the universal goniometer, when IMU sensors are placed in the frontal plane position.

**Figure 7:**
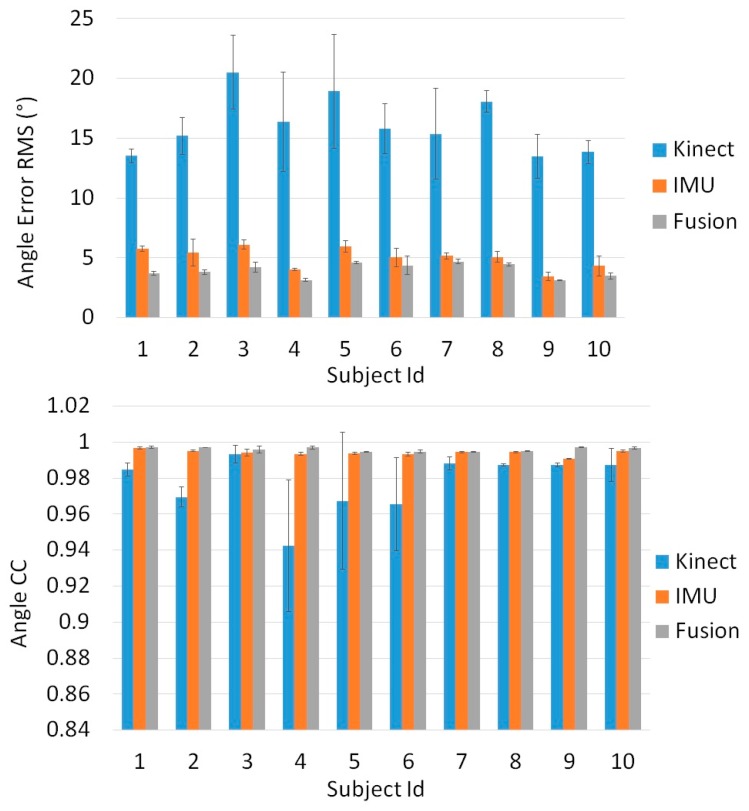
Angle error RMS (°) and angle Correlation Coefficient calculated for the Kinect, IMU and the proposed fusion algorithm Vs the universal goniometer, when IMU sensors are placed in the sagittal plane position.

**Figure 8:**
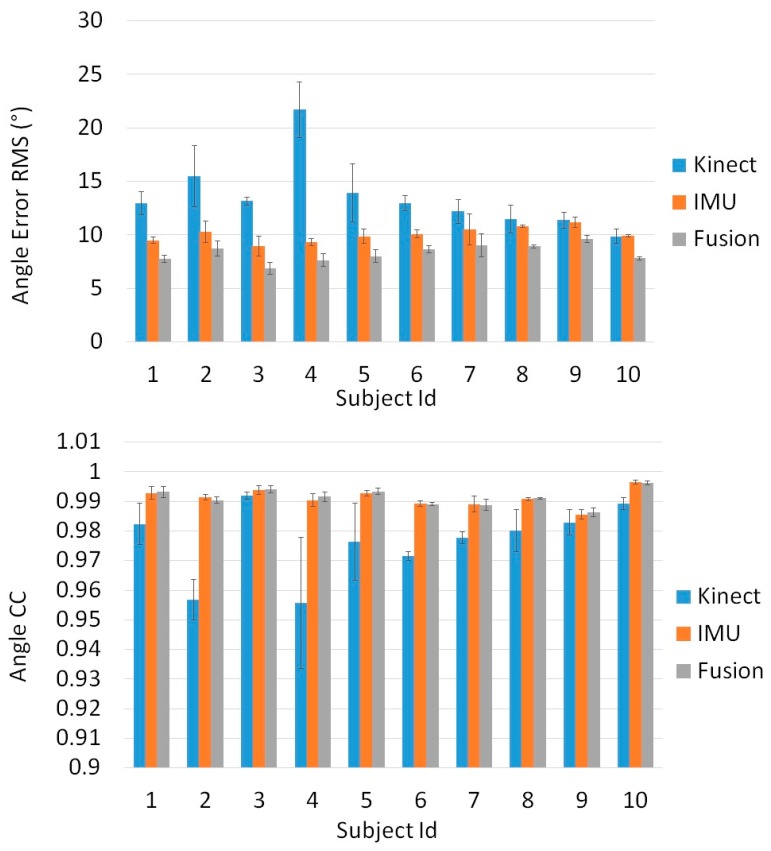
Angle error RMS (°) and angle Correlation Coefficient calculated for the Kinect, IMU and the proposed fusion algorithm Vs the universal goniometer, when IMU sensors are placed directly on the muscle.

**Figure 9:**
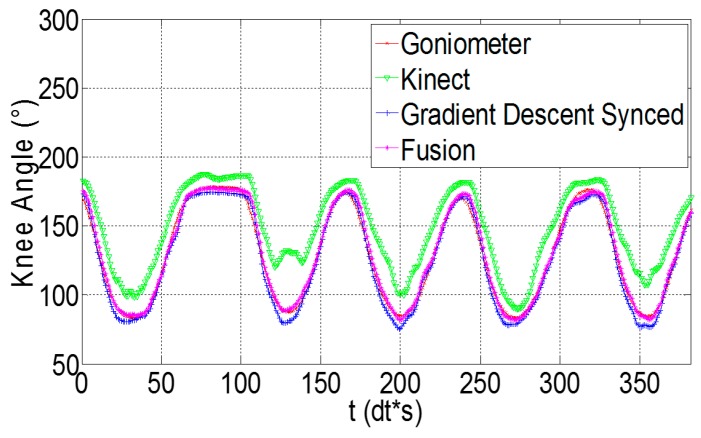
Knee angles estimated using goniometer, Kinect, gradient descent and sensor fusion, when IMU sensors are placed in the sagittal plane position.

Figure 6, Figure 7 and Figure 8 show the results of our real time, quaternion based, extended Kalman observer algorithm for fusion, for IMU sensors placed in the three proposed positions. Figure 9 presents the real time knee flexion angle estimation using the three different techniques. The fusion output shows a better estimation, when compared to IMU and Kinect outputs, for the three different IMU positions. When measuring the mean RMS error of the fusion output, we observed a decrease in the error compared to the same value obtained using IMUs, for all subjects and every IMU position. When using IMU sensors in the sagittal plane we obtained a decrease in the mean knee flexion angle error (mean of all 10 subjects over three trials each) using our fusion algorithm (3.96°) compared to the use of Kinect (14.76°) and IMU (5.04°). The proposed fusion also showed improvement in the angle error for IMUs placed in the frontal plane and on the muscle directly, however the error was slightly higher than those of the IMUs in the sagittal plane. This shows that the sagittal plane is the most accurate position to estimate the knee flexion angle. The correlation coefficient remained high for all of the tested estimation techniques, for every subject. Finally, the fusion output (in Figure 9) follows the goniometer signal, when IMU sensors are placed in the Sagittal plane position, and almost covers it, while the IMU and Kinect outputs are less precise. Statistical test (*t*-test, implemented in Matlab R2010b software (The MathWorks Inc., Natick, MA, USA)) showed a significant difference (*p* < 0.005) between the error from the Kinect and those from the IMU and Kinect-IMU. A significant difference (*p* < 0.005) was also noted between errors estimated from the IMU and Kinect-IMU fusion outcome.

## 4. Discussion

Motion capture sensors like Kinect or IMU are commonly used in exergames to track subject movements. The use of Kinect sensor has the advantages of portability and low cost which could lead to a home-based rehabilitation solution [4,6,7]. However, the lack of accuracy of joint kinematic estimation is one of the main obstacles for the use of this sensor in a clinical setting. This aspect is of great importance since medical experts, interested in analyzing joint angles, require a precision of six degrees for upper extremities [45] and 5.5 degrees for lower limbs [46]. Another important reason is related to the mathematical dependence between joint kinematics and muscle forces estimation [47]. Therefore, increasing the precision of joint angle estimation will lead to a more accurate data analysis and a more precise muscle force estimation. Thus, in this present study, we proposed a multi-sensor fusion scheme to improve the accuracy of knee joint kinematics. To these ends, a real time orientation-based extended Kalman algorithm was developed and tested.

The first analysis, where we attempted to synchronize two inertial sensors, proved that a lack of synchronization would lead to a significant total measurement error. It is important to note that the synchronization is a technical issue, necessary and required to improve the precision of sensor data acquisition and processing, in general for any system, and especially for real-time systems [41,48]. In this present study, a high variability (7.44°) between the synced and not synced angle estimations was observed. The high speed test further solidifies our point, since we discovered a high angle error (9.6°) that would be induced by a difference as small as one timestamp. Our synchronization algorithm proves to be effective in fixing de-synchronized samples and the difference between the samples used to calculate the angle is always at zero. Thus, one of the leading sources of error for our specific study, when coupling two inertial sensors at the same frequency rate, is caused by the lack of synchronization. In this present study, we proposed a synchronization scheme and the result shows a great improvement according to the test without synchronization.

In our second analysis, we tested the two chosen algorithms (Gradient Descent and extended Kalman) with and without synchronization against a universal goniometer. These algorithms already showed their robustness in many applications [22,24]. Table 2 shows that Gradient Descent with synchronization is the best algorithm to estimate the angle, with an RMS of 1.80° at slow speeds. Although, this RMS increases when repeating the same test at higher speeds, it is still better than the obtained values using other techniques. Moreover, the values of this RMS decreases when repeating the test with a fast followed by a slow motion, and is found to be close to values obtained in slow movements when only considering data registered during the slow phase. Finally, this experiment also highlights the success of our synchronization algorithm. Furthermore, it is clearly shown that in any speed, the Gradient Descent or extended Kalman synchronized algorithms are better at estimating the correct angle. This study allows us to eliminate extended Kalman orientation estimation and not synced algorithms, and thus Gradient Descent synced was the best algorithm.

The third analysis shows that muscle artefacts can add significant errors to the knee angle estimation. Following three tests with different sensor positions, we deduced that the sagittal plane is the least affected position by these artefacts. When comparing the sensors mounted directly on the thigh muscle with a goniometer, we obtain an error of 8.03° on knee angle estimation. This can be interpreted by the fact that the muscle’s flexion and extension is at its maximal range in that area of the thigh. Moreover, placing the sensor above the kneecap in the frontal plain yields an error of 4.75°, slightly higher than the 4.48° obtained in the sagittal plane. In another experiment, the error obtained from the Kinect’s angle estimation is dramatically higher (14.65°) than that of two sensors placed in the sagittal plane, and was compatible with values found in previous works that studied similar angle error using the Kinect [49,50,51]. Finally, we compared our fusion filter between two IMUs, mounted in three different positions, and a Kinect camera, against the goniometer. The fusion algorithm was tested on 10 subjects and the error behaviors between Kinect, IMU and Kinect-IMU solutions seem to be stable and similar over all subjects (see Figure 6, Figure 7 and Figure 8). The fusion output shows a greater resemblance to the goniometer signal, as it almost overlaps it in Figure 9. These results are also consistent since the fusion output gives a lower mean RMS angle error for all subjects, over different IMU position (Figure 6, Figure 7 and Figure 8). Both IMU and fusion results were acceptable with respect to the precision recommended by the experts [45,46], when considering IMUs in frontal or sagittal plane position. The results also showed that placing the IMUs in the sagittal plane gave the best estimation for the knee flexion angle.

In addition, according to available multi-sensor fusion schemes in the literature, we proposed one of the first orientation-based fusion schemes of visual and inertial sensors. Some previous works concentrated of determining joint positions through fusion between IMU and Kinect [32,35], while others were interested in some joint angles but did not compare estimated values to reference systems [36]. Our achieved angle estimation error can be compared to other works presented in Table 1 [33,34,37]. The study presented in [33] used no actual fusion between IMU and Kinect, as each sensor is used separately. This work helped remove Kinect limitations caused by occlusion of limbs, but failed to combine orientation measurements from both sensors, and compared instead knee angles calculated by IMUs and those calculated by the Kinect. The achieved error for knee angle estimation, while performing knee flexion, was 6.79° for left knee and 8.98° for right knee. Results in [34] showed a good position estimation for the upper body using a fusion approach between IMU and Kinect, however, the orientation estimation suffered from high errors. They measured the error of Euler angles relative to each bone (not joint), and found errors ranging from 1.71° to 24.64°. This error would become bigger when studying angles between two bones. Finally, study presented in [37] evaluated the system using four different movements with one subjects. Two of the movements had ranges of motion from zero to 90°, while the other two had hardly any motion. They concluded that their system showed a mean angle estimation error of 2.5° for elbow angle. However, they combined results from exercises with different movement characteristics, which cannot be done in order to obtain an objective estimation error. In summary, a quantitative comparison between our fusion outcomes with existing IMU-Kinect fusion methods [33,34,37] was performed. Both IMU and Kinect-IMU approaches achieved acceptable results, however our aim is to obtain the least error possible. Generally, the therapist requires more precision for a certain part of the body during a specific rehabilitation movement. The use of Kinect alone cannot provide this precision. Our strategy was to use IMU sensors on specific locations to achieve better precision. Thus, this fusion scheme helps to avoid the use of 10 additional IMU on the whole body.

Moreover, according to the related works on data fusion using Kinect and IMU sensors, existing studies investigated the use of IMU and Kinect fusion to estimate the position of joints and not their orientation while our study used the IMU and Kinect orientation data and angular velocity to estimate the orientation in the form of a 4D quaternion. Furthermore, our method described a detailed calculation of the filter covariance matrices. Finally, our system was designed as a real time orientation estimation system, while other systems obtain their fusion outputs through offline calculations.

## 5. Conclusions

This paper presents a new real time, quaternion based, extended Kalman observer for fusion between IMUs and Kinect sensors for knee angle estimation. We studied the different sources of error induced by IMUs and Kinect, and used this information to dynamically calculate a measurement correlation matrix for each specific sensor. We also proposed a synchronization approach, without the use of the sensor’s universal clock data, or request-response messages between PC and sensor. Our multi-sensor fusion approach showed a better precision compared to other approaches. In perspective, this fusion scheme will be applied to our home-based rehabilitation system [4,47].

## Figures and Tables

**Table 1:** Related work.

Study	Fusion Method	Fusion Input	Fusion Output	Targeted Application	Test
Feng et al. [32]	Multi-rate linear Kalman filter	Acceleration and computed quaternion data (IMU) and Kinect joint position	Joint position	Hand tracking	Raise hand, walk, lower hand (1 subject)
Destelle et al. [33]	Each system is used separately	Linear acceleration (IMU) and Kinect joint position	Joint position and angle	Whole body tracking	Knee flexion (1 subject)
Atrsaei et al. [34]	Unscented Kalman filter	Gyroscope and computed quaternion (IMU) and Kinect joint position and orientation	Joint position and angle	Upper body tracking	Arbitrary hand motion (1 subject)
Kalkbrenner et al. [35]	Linear Kalman filter	Unit orientation vectors (IMU) and Kinect joint position	Joint position	Upper body tracking	Shoulder abduction (10 subjects)
Tian et al. [36]	Unscented Kalman filter	Acceleration and magnetometer data (IMU) and Kinect joint position	Joint position and angle	Upper body tracking	Hand to mouth (1 subject)
Glonek et al. [37]	Weighted averaging	Computed quaternion (IMU) and Kinect joint position and orientation	Joint position and angle	Upper body tracking	Four tasks with different ranges of motion (1 subject)

**Table 2:** RMS (in °) and Correlation Coefficient (CC) between knee angles estimated using different algorithms Vs universal goniometer.

Algorithm Induced Errors									
Speed	Algorithm	Parameters							
		Mean (Angle Error RMS)	Std (Angle Error RMS)	Max (Angle Error RMS)	Min (Angle Error RMS)	Mean (Angle CC)	Std (Angle CC)	Max (Angle CC)	Min (Angle CC)
Slow (90°/s)	Kalman Synced	6.109	2.242	8.512	2.481	0.997	0.001	0.998	0.996
	Kalman Not Synced	7.665	2.012	9.839	4.957	0.993	0.003	0.995	0.986
	Gradient Descent Synced	1.805	0.383	2.288	1.29	0.999	0.0003	0.999	0.999
	Gradient Descent Not Synced	3.246	0.999	4.813	2.220	0.997	0.001	0.999	0.995
Fast (400°/s)	Kalman Synced	21.854	6.141	30.143	16.333	0.897	0.066	0.963	0.812
	Kalman Not Synced	31.546	6.507	37.089	22.291	0.753	0.108	0.910	0.677
	Gradient Descent Synced	9.414	2.650	12.993	6.709	0.980	0.014	0.996	0.960
	Gradient Descent Not Synced	22.095	6.432	27.217	12.690	0.873	0.068	0.972	0.813
Fast followed by Slow	Kalman Synced	10.847	2.283	12.207	8.210	0.976	0.006	0.983	0.971
	Kalman Not Synced	13.486	1.687	14.632	11.548	0.963	0.003	0.966	0.960
	Gradient Descent Synced	4.995	1.157	5.893	3.689	0.995	0.001	0.997	0.993
	Gradient Descent Not Synced	6.747	1.603	8.435	5.245	0.991	0.003	0.994	0.988

**Table 3:** RMS (in °) and Correlation Coefficient (CC) between knee angles estimated using different sensor positions Vs universal goniometer.

Sensor Position Errors									
Algorithm	Position	Parameters							
		Mean (Angle Error RMS)	Std (Angle Error RMS)	Max (Angle Error RMS)	Min (Angle Error RMS)	Mean (Angle CC)	Std (Angle CC)	Max (Angle CC)	Min (Angle CC)
Gradient Descent Synced	On the Muscle	8.030	1.159	8.965	6.732	0.995	0.001	0.997	0.995
	Frontal Plane	4.759	0.727	5.572	4.171	0.996	0.0005	0.997	0.996
	Sagittal Plane	4.481	0.869	5.661	3.654	0.997	0.001	0.998	0.995

**Table 4:** RMS (Angle in °, Angular Velocity in °/s) and Correlation Coefficient (CC) between knee angles and angular velocities estimated using Kinect Vs universal goniometer.

Kinect Errors								
Measured Quantity	Parameters							
	Mean (Error RMS)	Std (Error RMS)	Max (Error RMS)	Min (Error RMS)	Mean (CC)	Std (CC)	Max (CC)	Min (CC)
Angle	14.652	2.241	16.204	12.082	0.974	0.003	0.978	0.970
Angular Velocity	1.332	0.222	1.480	1.076	0.882	0.030	0.909	0.849

## References

[1] Hartley S., Ilagan V., Madden A., Posarac A., Seelman K., Shakespeare T., Sipos S., Swanson M., Thomas M., Qiu Z. (2011). World Report on Disability.

[2] Perry J.C., Andureu J., Cavallaro F.I., Veneman J., Carmien S., Keller T. (2010). Effective Game Use in Neurorehabilitation: User-Centered Perspectives. Handbook of Research on Improving Learning and Motivation through Educational Games.

[3] Chang Y.-J., Chen S.-F., Huang J.-D. (2011). A Kinect-based system for physical rehabilitation: A pilot study for young adults with motor disabilities. Res. Dev. Disabil..

[4] Tannous H., Istrate D., Ho Ba Tho M.C., Dao T.T. (2016). Serious game and functional rehabilitation for the lower limbs. Eur. Res. Telemed..

[5] Ibarra Zannatha J.M., Tamayo A.J.M., Sánchez Á.D.G., Delgado J.E.L., Cheu L.E.R., Arévalo W.A.S. (2013). Development of a system based on 3D vision, interactive virtual environments, ergonometric signals and a humanoid for stroke rehabilitation. Comput. Methods Programs Biomed..

[6] Chatzitofis A., Monaghan D., Mitchell E., Honohan F., Zarpalas D., O’Connor N.E., Daras P. (2015). HeartHealth: A Cardiovascular Disease Home-based Rehabilitation System. Procedia Comput. Sci..

[7] Lozano-Quilis J.-A., Gil-Gómez H., Gil-Gómez J.-A., Albiol-Pérez S., Palacios-Navarro G., Fardoun H.M., Mashat A.S. (2014). Virtual Rehabilitation for Multiple Sclerosis Using a Kinect-Based System: Randomized Controlled Trial. JMIR Serious Games.

[8] Sun T.-L., Lee C.-H. (2013). An Impact Study of the Design of Exergaming Parameters on Body Intensity from Objective and Gameplay-Based Player Experience Perspectives, Based on Balance Training Exergame. PLoS ONE.

[9] Paraskevopoulos I.T., Tsekleves E., Craig C., Whyatt C., Cosmas J. (2014). Design guidelines for developing customised serious games for Parkinson’s Disease rehabilitation using bespoke game sensors. Entertain. Comput..

[10] Cho S., Ku J., Cho Y.K., Kim I.Y., Kang Y.J., Jang D.P., Kim S.I. (2014). Development of virtual reality proprioceptive rehabilitation system for stroke patients. Comput. Methods Programs Biomed..

[11] Chen P.-Y., Wei S.-H., Hsieh W.-L., Cheen J.-R., Chen L.-K., Kao C.-L. (2012). Lower limb power rehabilitation (LLPR) using interactive video game for improvement of balance function in older people. Arch. Gerontol. Geriatr..

[12] Ding Q., Stevenson I.H., Wang N., Li W., Sun Y., Wang Q., Kording K., Wei K. (2013). Motion games improve balance control in stroke survivors: A preliminary study based on the principle of constraint-induced movement therapy. Displays.

[13] Jintrinix. www.jintronix.com/.

[14] Virtual-Reality. www.virtual-reality-rehabilitation.com/products/seeme.

[15] RespondWell. www.respondwell.com/.

[16] Williamson R., Andrews B.J. (2001). Detecting absolute human knee angle and angular velocity using accelerometers and rate gyroscopes. Med. Biol. Eng. Comput..

[17] Mayagoitia R.E., Nene A.V., Veltink P.H. (2002). Accelerometer and rate gyroscope measurement of kinematics: An inexpensive alternative to optical motion analysis systems. J. Biomech..

[18] Favre J., Jolles B.M., Aissaoui R., Aminian K. (2008). Ambulatory measurement of 3D knee joint angle. J. Biomech..

[19] Liu T., Inoue Y., Shibata K. (2009). Development of a wearable sensor system for quantitative gait analysis. Measurement.

[20] Pérez R., Costa Ú., Torrent M., Solana J., Opisso E., Cáceres C., Tormos J.M., Medina J., Gómez E.J. (2010). Upper Limb Portable Motion Analysis System Based on Inertial Technology for Neurorehabilitation Purposes. Sensors.

[21] Kalman R.E. (1960). A new approach to linear filtering and prediction problems. J. Basic Eng..

[22] Marins J.L., Yun X., Bachmann E.R., McGhee R.B., Zyda M.J. An Extended Kalman Filter for Quaternion-Based Orientation Estimation Using MARG Sensors. Proceedings of the International Conference on Intelligent Robots and Systems, 2001 IEEE/RSJ.

[23] Abyarjoo F., Barreto A., Cofino J., Ortega F.R. (2015). Implementing a Sensor Fusion Algorithm for 3D Orientation Detection with Inertial/Magnetic Sensors. Innovations and Advances in Computing, Informatics, Systems Sciences, Networking and Engineering.

[24] Madgwick S.O., Harrison A.J., Vaidyanathan R. Estimation of IMU and MARG Orientation Using a Gradient Descent Algorithm. Proceedings of the 2011 IEEE International Conference on Rehabilitation Robotics (ICORR).

[25] ShimmerSensing. www.shimmersensing.com/.

[26] Miezal M., Taetz B., Bleser G. (2016). On Inertial Body Tracking in the Presence of Model Calibration Errors. Sensors.

[27] Dejnabadi H., Jolles B.M., Aminian K. (2005). A New Approach to Accurate Measurement of Uniaxial Joint Angles Based on a Combination of Accelerometers and Gyroscopes. IEEE Trans. Biomed. Eng..

[28] Dejnabadi H., Jolles B.M., Casanova E., Fua P., Aminian K. (2006). Estimation and Visualization of Sagittal Kinematics of Lower Limbs Orientation Using Body-Fixed Sensors. IEEE Trans. Biomed. Eng..

[29] Favre J., Aissaoui R., Jolles B.M., de Guise J.A., Aminian K. (2009). Functional calibration procedure for 3D knee joint angle description using inertial sensors. J. Biomech..

[30] Bouvier B., Duprey S., Claudon L., Dumas R., Savescu A. (2015). Upper Limb Kinematics Using Inertial and Magnetic Sensors: Comparison of Sensor-to-Segment Calibrations. Sensors.

[31] Zhang L., Sturm J., Cremers D., Lee D. Real-Time Human Motion Tracking Using Multiple Depth Cameras. Proceedings of the 2012 IEEE/RSJ International Conference on Intelligent Robots and Systems.

[32] Feng S., Murray-Smith R. (2014). Fusing Kinect Sensor and Inertial Sensors with Multi-Rate Kalman Filter. Proceedings of the IET Conference on Data Fusion & Target Tracking 2014: Algorithms and Applications (DF&TT 2014).

[33] Destelle F., Ahmadi A., O’Connor N.E., Moran K., Chatzitofis A., Zarpalas D., Daras P. Low-Cost Accurate Skeleton Tracking Based on Fusion of Kinect and Wearable Inertial Sensors. Proceedings of the 22nd European Signal Processing Conference (EUSIPCO).

[34] Atrsaei A., Salarieh H., Alasty A. (2016). Human Arm Motion Tracking by Orientation-Based Fusion of Inertial Sensors and Kinect Using Unscented Kalman Filter. J. Biomech. Eng..

[35] Kalkbrenner C., Hacker S., Algorri M., Blechschmidt-trapp R. Motion Capturing with Inertial Measurement Units and Kinect—Tracking of Limb Movement using Optical and Orientation Information. Proceedings of the International Conference on Biomedical Electronics and Devices.

[36] Tian Y., Meng X., Tao D., Liu D., Feng C. (2015). Upper limb motion tracking with the integration of IMU and Kinect. Neurocomputing.

[37] Glonek G., Wojciechowski A. (2016). Hybrid Method of Human Limb Joints Positioning—Hand Movement Case Study. Information Technologies in Medicine.

[38] Tannous H., Istrate D., Ho Ba Tho M.C., Dao T.T. (2016). Feasibility study of a serious game based on Kinect system for functional rehabilitation of the lower limbs. Eur. Res. Telemed..

[39] NASA Mission Planning and Analysis Division Euler Angles, Quaternions, and Transformation Matrices. www.ntrs.nasa.gov/archive/nasa/casi.ntrs.nasa.gov/19770024290.pdf.

[40] Chen S., Brantley J.S., Kim T., Ridenour S.A., Lach J. (2013). Characterising and minimising sources of error in inertial body sensor networks. Int. J. Auton. Adap. Commun. Syst..

[41] Wåhslén J., Orhan I., Lindh T. (2011). Local Time Synchronization in Bluetooth Piconets for Data Fusion Using Mobile Phones.

[42] Davis R.B., Ounpuu S., Tyburski D., Gage J.R. (1991). A gait analysis data collection and reduction technique. Hum. Mov. Sci..

[43] Cleland I., Kikhia B., Nugent C., Boytsov A., Hallberg J., Synnes K., McClean S., Finlay D. (2013). Optimal Placement of Accelerometers for the Detection of Everyday Activities. Sensors.

[44] Wang L., Zhang Z., Sun P. (2015). Quaternion-based Kalman filter for AHRS using an adaptive-step gradient descent algorithm. Int. J. Adv. Robot. Syst..

[45] Gajdosik R.L., Bohannon R.W. (1987). Clinical measurement of range of motion review of goniometry emphasizing reliability and validity. Phys. Ther..

[46] Brosseau L., Tousignant M., Budd J., Chartier N., Duciaume L., Plamondon S., O’Sullivan J.P., O’Donoghue S., Balmer S. (1997). Intratester and intertester reliability and criterion validity of the parallelogram and universal goniometers for active knee flexion in health. Physiother. Res. Int..

[47] Dao T.T., Tannous H., Pouletaut P., Gamet D., Istrate D., Ho Ba Tho M.C. (2016). Interactive and Connected Rehabilitation Systems for E-Health. IRBM.

[48] Olson E. A Passive Solution to the Sensor Synchronization Problem. Proceedings of the 2010 IEEE/RSJ International Conference on Intelligent Robots and Systems.

[49] Bonnechère B., Jansen B., Salvia P., Bouzahouene H., Omelina L., Moiseev F., Sholukha V., Cornelis J., Rooze M., Van Sint Jan S. (2014). Validity and reliability of the Kinect within functional assessment activities: Comparison with standard stereophotogrammetry. Gait Posture.

[50] Pfister A., West A.M., Bronner S., Noah J.A. (2014). Comparative abilities of Microsoft Kinect and Vicon 3D motion capture for gait analysis. J. Med. Eng. Technol..

[51] Plantard P., Auvinet E., Pierres A.-S., Multon F. (2015). Pose Estimation with a Kinect for Ergonomic Studies: Evaluation of the Accuracy Using a Virtual Mannequin. Sensors.

